# Association of mushroom consumption with all-cause and cause-specific mortality among American adults: prospective cohort study findings from NHANES III

**DOI:** 10.1186/s12937-021-00691-8

**Published:** 2021-04-22

**Authors:** Djibril M. Ba, Xiang Gao, Joshua Muscat, Laila Al-Shaar, Vernon Chinchilli, Xinyuan Zhang, Paddy Ssentongo, Robert B. Beelman, John P. Richie

**Affiliations:** 1 Department of Public Health Sciences, Penn State College of Medicine, Hershey, PA USA; 2 Department of Nutritional Sciences, The Pennsylvania State University, University Park, PA USA; 3 Department of Food Science and Center for Plant and Mushroom Foods for Health, College of Agricultural Sciences, The Pennsylvania State University, University Park, PA USA

**Keywords:** Mushroom, Mortality risk, Diet, Prospective study, NHANES III

## Abstract

**Background:**

Whether mushroom consumption, which is rich in several bioactive compounds, including the crucial antioxidants ergothioneine and glutathione, is inversely associated with low all-cause and cause-specific mortality remains uncertain**.** This study aimed to prospectively investigate the association between mushroom consumption and all-cause and cause-specific mortality risk.

**Methods:**

Longitudinal analyses of participants from the Third National Health and Nutrition Examination Survey (NHANES III) extant data (1988–1994). Mushroom intake was assessed by a single 24-h dietary recall using the US Department of Agriculture food codes for recipe foods. All-cause and cause-specific mortality were assessed in all participants linked to the National Death Index mortality data (1988–2015). We used Cox proportional hazards regression models to calculate multivariable-adjusted hazard ratios (HRs) and 95% confidence intervals (95% CIs) for all-cause and cause-specific mortality.

**Results:**

Among 15,546 participants included in the current analysis, the mean (SE) age was  44.3 (0.5) years. During a mean (SD) follow-up duration of 19.5 (7.4) years , a total of 5826 deaths were documented. Participants who reported consuming mushrooms had lower risk of all-cause mortality compared with those without mushroom intake (adjusted hazard ratio (HR) = 0.84; 95% CI: 0.73–0.98) after adjusting for demographic, major lifestyle factors, overall diet quality, and other dietary factors including total energy. When cause-specific mortality was examined, we did not observe any statistically significant associations with mushroom consumption. Consuming 1-serving of mushrooms per day instead of 1-serving of processed or red meats was associated with lower risk of all-cause mortality (adjusted HR = 0.65; 95% CI: 0.50–0.84). We also observed a dose-response relationship between higher mushroom consumption and lower risk of all-cause mortality (*P-*trend = 0.03).

**Conclusion:**

Mushroom consumption was associated with a lower risk of total mortality in this nationally representative sample of US adults.

**Supplementary Information:**

The online version contains supplementary material available at 10.1186/s12937-021-00691-8.

## Background

Mushrooms are rich in micronutrients and may represent an important component of a healthy diet. However, their unique nutritional values have not been fully appreciated. Although mushrooms share some nutritional characteristics with plant-derived foods that are thought to be rich in antioxidants, they biologically belong to the fungal kingdom [1, 2]. They have been consumed by humans for centuries because mushrooms are low in calories, sodium, and fats, yet the US commercial mushroom industry did not take hold until the early 1900s in Pennsylvania [3–6]. Despite the increase of mushroom consumption over the years [7], retail per capita consumption of mushrooms in the US was still relatively low compared to other countries worldwide, while the consumption of meat and other animal-derived food sources were consistently high. According to the US Department of Agriculture (USDA) National Agricultural Statistics Service, fresh white mushroom per capita consumption is slightly less than 3 pounds per year in the US [2].

Mushrooms are also rich in bioactive compounds, including fiber, polysaccharides such as β-glucans [8], selenium [9, 10], vitamins [11], and the crucial sulfur-containing antioxidants ergothioneine and glutathione which are thought to play significant roles in the prevention of chronic diseases and premature death and promotion of healthy aging [12–14]. Ergothioneine levels differ by mushroom types with shiitake, oyster, and maitake mushrooms, which are widely consumed in Eastern countries, being higher compared to Agaricus bisporus species such as white button, crimini, and portabella mushrooms, which are predominantly distributed and consumed in the US [1, 14]. Recently, the preventive properties of mushroom extracts and their constituent bioactive agents have gained considerable research attention around the world [3, 5, 15].

Some epidemiological studies have reported associations between mushroom consumption and low risks of chronic diseases, such as cancers [16, 17], metabolic syndrome [18], cognitive impairment [19], and dementia [20] although some studies failed to observe significant associations [21–25]. However, whether the consumption of mushrooms is associated with better survival and low risk of premature mortality remains uncertain using large-scale epidemiologic studies. A previous systematic review and meta-analysis of fruit and vegetable intake found no association between mushroom consumption and all-cause mortality [26]. However, their meta-analysis only included two studies where the mushroom intake was estimated and these were mainly focused on vegetable subtypes including mushrooms, and, consequently, subject to potential misclassification bias [27, 28]. We thus aim to investigate the association between mushroom consumption and the risk of all-cause and cause-specific mortality by conducting prospective analyses using a nationally representative dataset from the Third National Health and Nutrition Examination Survey (NHANES III) (1988–1994).

## Methods

### Data source

We conducted a prospective cohort study using the public released de-identified NHANES III (1988–1994). All-cause and cause-specific mortality were assessed in all participants linked to the National Death Index (NDI) mortality data (1988–2015). The NHANES III study was conducted by the National Center for Health Statistics (NCHS) of the Centers for Disease Control and Prevention (CDC) and employed a complex, multistage, probability sampling design that allows results to be extrapolated to the entire US population. The program is designed to examine the health and nutritional status of the US civilian, non-institutionalized population aged 2 months and older for NHANES III participants [29]. Details on the NHANES Laboratory/Medical Technologists Procedures and Anthropometry Procedures are described elsewhere [30]. The survey protocol was approved annually by the NCHS Research Ethics Review Board and all participants provided written informed consent [29]. Detailed information about the dietary interview portion has been published previously [31].

Since NHANES data are de-identified and publicly available data, the Institutional Review Board (IRB) at the researchers’ institution does not consider this to be human subject research. Therefore, human subjects’ approval was not necessary nor sought since this was a de-identified data-only study.

### Study population

**Fig. 1:**
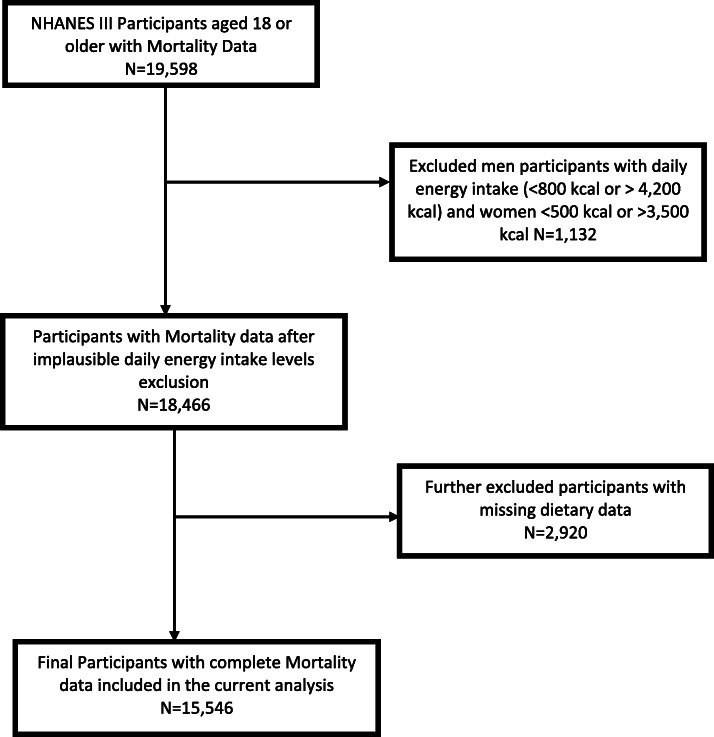
Study participant flowchart

The current study included individuals aged 18 years or older from a nationally representative sample of NHANES III with data on mortality status (*n* = 19,598). As done by a previous study [32], participants who reported implausible daily energy intake levels (< 800 kcal or > 4200 kcal for men and < 500 kcal or > 3500 kcal for women) (*n* = 1132) and  participants with missing dietary data (*n* = 2920) were excluded, leaving 15,546 participants in the current analysis for NHANES III participants (Fig. 1).

### Assessment of mushroom consumption

Mushroom intake was estimated based on NHANES III dietary intake data obtained via a single 24-h recall obtained by a trained interviewer with the use of an automated, microcomputer-based dietary interview and coding system known as the NHANES III Dietary Data Collection (DDC) System [31, 33]. All eligible participants provided a single 24-h dietary recall and a small subsample of approximately 8% of participants was eligible for a second 24-h dietary recall [29]. The USDA Survey Nutrient Database System (SNDB) was used to determine the nutrient content of foods. The NHANES III Individual Foods File (IFF) contains USDA food codes for recipe foods and the amount eaten in grams for every food item (identified by a unique 7-digit code) and was searched to identify each food containing mushrooms. Detailed information about NHANES III dietary data collection and IFF can be found on the NHANES website (https://wwwn.cdc.gov/nchs/data/nhanes3/2a/iff-acc.pdf). As done by a previous study [34], mushroom consumption was calculated based on the intake of foods that were mostly mushrooms or mushrooms alone, for example egg omelet or scramble egg served with mushrooms, or dishes with mushrooms as a recipe component, for example mushrooms gravy*.* In the mixed foods with mushrooms, the US Environmental Protection Agency-USDA Food Commodity Intake Database (FCID) commodity codes were used to determine the actual amounts of mushroom intake as follow: grams of intake by USDA food code time the commodity weight of mushroom contribution per 100 g of the USDA food code [34]. Details information regarding the Food Commodity Intake Database is described elsewhere [35]. Only individuals with reliable and complete dietary records for mushroom intake as determined by NCHS were included in the current analysis. Unique USDA food codes used to identify mushroom consumers (*n* = 544) are presented in the supplemental Table 1.

### Mortality ascertainment

The endpoints for this study were all-cause and cause-specific mortality, ascertained by NCHS using death certificates. The de-identified and anonymized data of the NHANES III participants were linked to NDI Mortality Files (*n* = 5826) with a probabilistic matching algorithm to determine mortality status using the NHANES III sequence number. The NCHS public-use linked mortality file provides mortality follow-up data from the date of NHANES III survey participation up until December 31, 2015 (1988–2015) [36]. Participants with no matched death record were considered to be alive during the entire follow-up period.

All cause-mortality in the current analysis included all specified causes of death recorded in the Public-use Linked Mortality files. Cause-of-mortality coding for all US mortality occurring prior to 1999 was determined using the Ninth Revision of the International Classification of Diseases (ICD-9), while for all mortality after 1998 follows the Tenth Revision of the International Classification of Diseases, (ICD-10) for mortality occurring in or after 1999. To facilitate and assist researchers with analyses, the NCHS recoded all mortality occurring prior to 1999 coded under ICD-9 guidelines into comparable ICD-10 according to the underlying cause of mortality groups [36]. All specified causes of mortality as well as underlying causes of mortality were recorded in the Public-use Linked Mortality files using the following ICD-10 codes: Cardiovascular diseases including heart diseases (I00-I09, I11, I13, I20-I51) and cerebrovascular diseases (I60-I69), malignant neoplasms. (C00-C97). Other cause-specific mortality included: chronic lower respiratory diseases (J40-J47), accidents (unintentional injuries) (V01-X59, Y85-Y86), Alzheimer’s disease (G30), diabetes mellitus (E10-E14), influenza and pneumonia (J09-J18), nephritis, nephrotic syndrome, and nephrosis (N00-N07, N17-N19, N25-N27) and residual causes.

### Assessment of dietary intakes and covariates

For the present analysis, the following covariates were extracted from the existing NHANES III 24-h recall dietary intake datasets based on previous literature [25, 34]**:** intake of total energy (kcal/d), alcohol (g/d), energy-adjusted fat (g)/1000 kcal/d), carbohydrates (g)/1000 kcal/d), fiber (g)/1000 kcal/d), and the Healthy Eating Index (HEI-2000), a measure of overall diet quality, which was included in the NHANES III data. To compute the HEI-2000 for NHANES III participants, Food Guide Pyramid serving sizes recommended by the USDA was applied to the food servings obtained through a 24-h [37]. The HEI-2000 includes a 10-component system of five food groups including fruits, vegetables, four nutrients, and a measure of variety in food intake. The total score ranges from 0 to 100, with a higher score suggesting a healthier diet [38]. Information on age (years), sex (men/women), ethnicity-race (non-Hispanic white, non-Hispanic Black, Mexican American, others), US regions (Northeast, Midwest, South, West), place of residence (Urban/Rural), education attainment (years), marital status (married, widowed/divorced/separated, never married), smoking status (smoked  100+ cigarettes in life yes or no), and physical activity level (moderate to vigorous) were collected through self-reported. The body measurement (including weight and height) was measured at the time of physical examination in a mobile examination center (MEC) or in the participant’s home. The body mass index (BMI) was calculated as weight in kilograms divided by height in meters squared and was categorized into 5 groups using CDC classification: underweight (< 18.5 kg/m^2^), normal weight (18.5–24.9), overweight (25.0–29.9), obese (30.0–34.9), and excessively obese (≥35.0). Given the small number of participants in the first and the last categories, BMI was later categorized into 3 groups: normal weight (< 24.9), overweight (25.0–29.9), obese (≥30).

### Statistical analysis

SAS statistical software version 9.4 (SAS Institute) was used to perform all statistical analyses using 2-sided *P* < .05 as the significance level. Survey analysis procedures were used to account for the sample weights, clustering, and stratification of the complex sampling design as specified in the instructions for using NHANES data to ensure nationally representative estimates [39]. Univariate analyses were conducted to assess the statistical significance of differences in weighted percentages for categorical variables using the Rao-Scott χ2 test and weighted means for continuous variables using t-test. For each participant, mortality follow-up time was calculated as the time from the baseline survey participation interview date until the date of death or end of follow-up (December 31, 2015), whichever came first. The consumption of mushrooms was deemed as the primary exposure during the study period. We used time-dependent multivariable Cox proportional hazards models to assess the association of mushroom consumption with all-cause and cause-specific mortality risk during the follow-up. The Cox proportional hazards regression models were performed to calculate multivariable-adjusted hazard ratios (HRs) and 95% confidence intervals (95% CIs) for all-cause and cause-specific mortality and the proportional hazards assumption was not violated. The following potential confounders were controlled for in the multivariable Cox regression models: age (years), sex (men/women), ethnicity-race (non-Hispanic white, non-Hispanic Black, Mexican American, others) region (Northeast, Midwest, South, West), place of residence (rural/urban), education attainment (years), marital status (categorical), BMI (categorical), moderate to vigorous physical activity (yes vs. no), smoking (smoked 100+ cigarettes in life, yes vs. no) intake of total energy (kcal/d), alcohol intake (g/d), energy-adjusted fat (g)/1000 kcal/d), carbohydrate (g)/1000 kcal/d), fiber (g)/1000 kcal/d), and the HEI-2000. To further examine whether there was  evidence of a dose-response relationship between greater mushroom consumption and all-cause mortality risk, we further categorized mushroom intake into 4 categories: no mushroom intake (0 g/d, *n* = 15,002), lowest (median intake = 10 g/d, range = 23.9, *n* = 346), middle (median intake = 35 g/d, range = 19.0, *n* = 104), and high (median intake = 72 g/d, range = 141.4, *n* = 94). Test for linear trend was examined for significance by using the median value for each category of mushroom intake, which was analyzed as a continuous variable in the multivariable-adjusted Cox model as done by previous researchers [40]. We did a single imputation using the fully conditional specification method for missing values for demographic and lifestyle variables [41]. As a secondary analysis, we conducted a nutritional substitution analysis to compare the health effect of substituting 1-serving/d of mushroom for 1-serving/d of red or processed meat. As done by a previous study, 1-serving of red or processed meat was defined as 3.5-oz equivalents and 1-serving of mushroom as 70 g [42, 43]. Red or processed meat includes such as beef, veal, pork, lamb, cured, and organs meat. The association of substituting 1-serving/d of mushroom for 1-serving/d of red or processed meat with all-cause mortality was examined by including both as continuous variables in the same multivariable Cox regression model adjusting for age, sex, ethnicity-race, region, place of residence, education attainment (years), marital status, BMI, moderate to vigorous physical activity (yes vs. no, smoking ( smoked 100+ cigarettes in life yes vs. no), alcohol intake (g/d), total energy (kcal/d), and other dietary variables, including poultry (oz/d), fish (oz/d), eggs (oz/d), nuts/soy (oz/d), legumes (svg/d), fruit (svg/d), dark green/ yellow vegetables (svg/d), dairyy (svg/d), discretionary fat (g/d), and added sugar (tsp/d). The difference in their regression coefficients, variances, and covariance were used to estimate the HR and 95% CIs for the substitution effect. This methodology has been widely used in providing a better solution to dietary patterns [25, 44, 45].

To further test for the robustness of our results, we conducted a series of sensitivity analyses. First, to minimize potential bias, we further adjusted for a propensity score , which was calculated by including the aforementioned covariates in the final model 3. This approach allows us to balance baseline data between participants with mushroom intake and those without mushroom intake.

Second, to understand the short- vs. long-term impact of mushroom intake on mortality, we dichotomously calculated hazard by excluding when mortality cases occurred during the first 2 years of follow-up, adjusting for preceding covariates. Third, because major chronic diseases are strongly associated with the risk of mortality [46], we conducted a sensitivity analysis by excluding participants with baseline congestive heart failure or hypertension/high blood pressure, cancer, diabetes, or changed their diet because of high blood pressure. Fourth, the interaction between mushroom intake and age, ethnicity-race, sex in association with all-cause mortality were statistically tested by including the interaction terms in the Cox regression model. Lastly, a previous study of NHANES data suggested that mushroom intake was associated with better nutrients intake including micronutrients and diet quality [34], therefore we further adjusted our final model 3 for energy-adjusted Vitamin E (mg)/1000 kcal/d), β-carotene (mcg)/1000 kcal/d), vitamin C (mg)/1000 kcal/d), copper (mg)/1000 kcal/d), and selenium (mcg)/1000 kcal/d) intake.

## Results

**Fig. 2:**
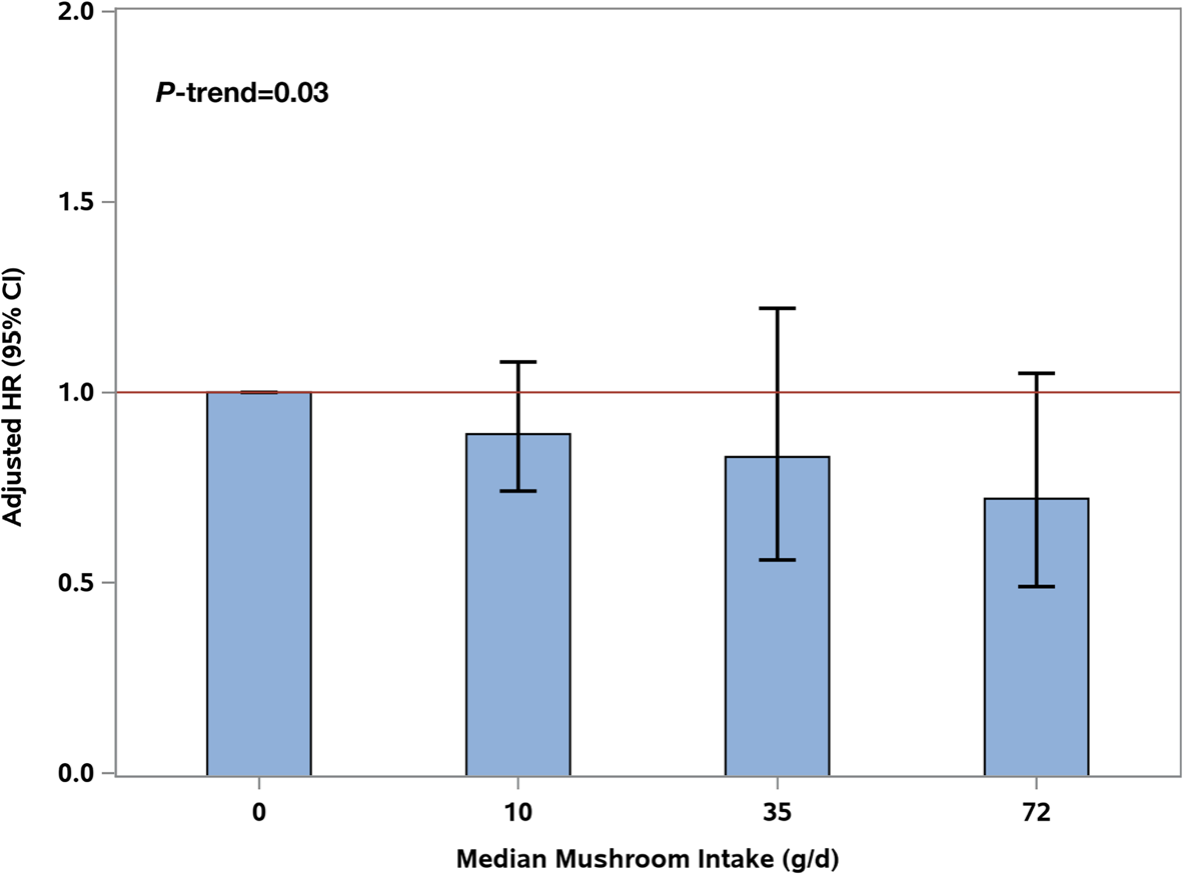
Model 3 Adjusted Hazard Ratios for Mushroom Intake Across Groups

A total of 15,546 participants (the mean age 44.3 ± 0.5 y) were included in the current analysis. More than half of the study participants were women 8499 (54.1%); 6368 participants (76.2%) were non-Hispanic white (Table 1). Compared with individuals without mushroom intake, a higher proportion of mushroom consumers were from the South region of the US, non-Hispanic whites, and had higher education attended (Table 1). The mean HEI-2000 was higher among individuals who consumed mushrooms compared to non-mushroom consumers (Table 1). During a mean 19.5 ± 7.4 y of follow-up (303,669 person-years), we identified a total of 5826 mortality cases. In the age- and sex-adjusted model (model 1), individuals with mushroom consumption had lower risk of all-cause mortality compared with those without mushroom consumption (adjusted hazard ratio (HR) = 0.79; 95% CI: 0.67–0.92; Table 2). After additional adjustment for other potential confounding factors (model 3), including ethnicity-race, region, place of residence, education status, marital status, BMI, physical activity, total energy intake, fats, carbohydrates, fiber, smoking status, alcohol intake, and the HEI-2000, the association between mushroom consumption and all-cause mortality remained statistically significant (adjusted HR = 0.84; 95% CI: 0.73–0.98; Table 2). When mushroom intake was further divided into 4 groups (Fig. 2), we observed a significant dose-response relationship between greater mushroom consumption and lower risk of all-cause mortality (*P-*trend = 0.03).

**Table 1:** Weighted baseline characteristics of the study participants, NHANES III (*N* = 15,546)

	Participants, No.		
Characteristic	No Mushroom Intake (***n*** = 15,002)	Mushroom Intake (***n*** = 544)	***P*** value‡
Age, mean (SE)^**a**^, years	44.4 ± 0.4	42.9 ± 1.4	0.25
Gender %			0.90
Men	6824 (45.8)	223 (46.3)	
Women	8178 (54.2)	321 (53.7)	
Regions of United States (%) ^**b**^			0.15
Northeast	2050 (20.8)	66 (16.8)	
Midwest	2897 (24.1)	109 (22.3)	
South	6491 (34.0)	209 (33.4)	
West	3564 (21.1)	160 (27.5)	
Place of residence %			0.31
Urban	7325 (48.2)	267 (51.9)	
Rural	7677 (51.8)	277 (48.1)	
Race-Ethnicity %			<0.0001
Non-Hispanic White	6039 (75.6)	329 (87.1)	
Non-Hispanic Black	4214 (11.2)	82 (4.8)	
Mexican American	4149 (5.3)	114 (3.1)	
Others	600 (8.0)	19 (5.0)	
Education attainment, years	12.2 ± 0.1	13.4 ± 0.2	<0.0001
Marital status %			0.22
Married	8979 (64.4)	349 (66.7)	
Widowed/Divorced/Separated	3208 (17.6)	95 (13.4)	
Never married	2815 (17.9)	100 (19.9)	
Body mass index (kg/m^2^) %			0.13
< 24.9	6063 (45.9)	224 (43.8)	
25.0–29.9	5118 (31.7)	201 (37.6)	
≥ 30.0	3821 (22.4)	119 (18.6)	
Moderate to vigorous activity%	5364 (39.8)	221 (47.4)	0.007
Smoked 100+ cigarettes %	7334 (52.7)	257 (49.6)	0.23
Alcohol intake, g/d	8.6 ± 0.5	10.5 ± 1.3	0.19
Nutrients Intakes			
Energy intake, kcal/d	2040.3 ± 11.3	2215.1 ± 42.1	0.0002
Fiber intake, (g)/1000 kcal/d	8.1 ± 0.1	8.6 ± 0.2	0.04
Fat intake, (g)/1000 kcal/d	37.0 ± 0.2	39.1 ± 0.6	0.001
Carbohydrate intake, (g)/1000 kcal/d	125.4 ± 0.7	118.4 ± 1.7	0.0002
Healthy Eating Index-2000	63.8 ± 0.3	66.3 ± 0.8	0.001
Antioxidant micronutrients			
Vitamin E (mg)/1000 kcal/d)	4.4 ± 0.1	4.8 ± 0.2	0.01
β-carotene (mcg)/1000 kcal/d)	1480.0 ± 38.2	1637.4 ± 117.6	0.22
Vitamin C (mg)/1000 kcal/d)	52.6 ± 0.8	60.8 ± 3.5	0.03
Copper (mg)/1000 kcal/d)	0.6 ± 0.01	0.7 ± 0.02	<0.0001
Selenium (mcg)/1000 kcal/d)	56.3 ± 0.4	59.6 ± 1.7	0.05

^a, b^ All percentages and means ± SE are weighted for complex survey design to be nationally representative estimates. The focus should be on the survey-weighted proportions and means ± SE

‡For categorical variables, *P*-value was calculated by the Rao-Scott χ2 test. For continuous variables, *P*-value was calculated using a t-test

*SE:* Standard Error

**Table 2:** Adjusted Hazard Ratios (95% confidence intervals) of Mortality according to baseline mushroom intake status among 15,546 NHANES III participants

	No Mushroom Intake	Mushroom Intake
Person year (PY)	292,296	11,373
Mortality case #	5657	169
Incidence rate (95% CI), per 1000 PY	19.4 (18.9, 20.0)	14.9 (12.8, 17.3)
Model 1	1(ref)	0.79 (0.67, 0.92)
Model 2	1(ref)	0.85 (0.73, 0.97)
Model 3	1(ref)	0.84 (0.73, 0.98)
**Sensitivity analysis**^**a**^		
Propensity score adjustment	1(ref)	0.86 (0.74, 0.99)
Excluding 385 deaths during the first 2 years of follow-up	1(ref)	0.82 (0.70, 0.97)
Excluding 4826 participants with major chronic diseases^**b**^	1(ref)	0.83 (0.65, 1.06)

Model 1: Age (years) and sex (men/women) adjusted

Model 2: Model 1 + ethnicity-race (non-Hispanic White, Non-Hispanic Black, Mexican American, others), US regions (Northeast, Midwest, South, West), place of residence (urban/rural), education attainment (years), marital status (married, widowed/divorced/separated, never married) adjusted

Model 3: Model 2 + further adjustment of BMI (< 24.9, 25.0–29.9, ≥30), moderate to vigorous physical activity (yes/no), alcohol (g/d), smoked 100+ cigarettes in life (yes/no), total energy intake (kcal/d), fat (g)/1000 kcal/d), carbohydrates (g)/1000 kcal/d), fiber (g)/1000 kcal/d), and Healthy Eating Index-2000 score

Further adjustment of antioxidant micronutrients: Model 3 + further adjustment of Vitamin E (mg)/1000 kcal/d), β-carotene (mcg)/1000 kcal/d), vitamin C (mg)/1000 kcal/d), copper (mg)/1000 kcal/d), and selenium (mcg)/1000 kcal/d) intake did not change the final the Hazard Ratios and the 95% CIs from Model 3

^a^Based on model 3

^b^Major chronic diseases include congestive heart failure, hypertension/high blood pressure, diabetes, cancer or changed their diet because of high blood pressure

When cause-specific mortality was examined, we did not observe any significant associations with mushroom consumption. The adjusted HRs were reported in Table 3 as follow: cardiovascular disease (adjusted HR = 0.82; 95% CI: 0.56–1.21), cancer (adjusted HR = 0.77; 95% CI: 0.50–1.19), Alzheimer disease (adjusted HR = 0.90; 95% CI: 0.31–2.60), diabetes mellitus (adjusted HR = 0.32; 95% CI: 0.06–1.65), and other causes of mortality (adjusted HR = 0.93; 95% CI: 0.70–1.23).

**Table 3:** Adjusted Hazard Ratios (95% confidence intervals) of cause-specific Mortality according to baseline mushroom intake status among 15,546 participants

Individual Mortality Cause	Total # cases	No Mushroom Intake	Mushroom Intake
Cardiovascular diseases	1764	1(ref)	0.82 (0.56, 1.21)
Cancer	1235	1(ref)	0.77 (0.50, 1.19)
Alzheimer	140	1 (ref)	0.90 (0.31, 2.60)
Diabetes mellitus	225	1 (ref)	0.32 (0.06, 1.65)
^a^Other causes of Mortality	2407	1(ref)	0.93 (0.70, 1.23)

Model 3: age (years), sex (male/female), ethnicity-race (non-Hispanic White, Non-Hispanic Black, Mexican American, others), US regions (Northeast, Midwest, South, West), place of residence (urban/rural), education attainment (years), marital status (married, widowed/divorced/separated, never married), BMI (< 24.9, 25.0–29.9, ≥30), moderate to vigorous physical activity (yes/no), alcohol (g/d), smoked 100+ cigarette in life (yes/no), total energy intake (kcal/d), fat (g)/1000 kcal/d), carbohydrates (g)/1000 kcal/d), fiber (g)/1000 kcal/d), and Healthy Eating Index-2000 score

^a^Other reported cause-specific mortality included: chronic lower respiratory diseases, accidents (unintentional injuries), influenza and pneumonia, nephritis, nephrotic syndrome, and nephrosis and residual causes

In the nutritional substitution model, replacing red or processed meat with mushrooms was associated with lower risk of mortality. The adjusted HR for all-cause mortality was 0.65 (95% CI: 0.50–0.84) when 1-serving/d of red or processed meat was substituted for an equivalent amount of 1-serving/d of mushroom.With further adjustment of a propensity score in the final model , the association between mushroom intake and all cause-mortality was attenuated but remained significant (adjusted HR = 0.86; 95% CI: 0.74–0.99; Table 2). Exclusion of 385 mortality cases occurring during the first 2 years of follow-up generated similar results (adjusted HR = 0.82; 95%CI: 0.70–0.97; Table 2). Further, exclusion of participants with congestive heart failure or hypertension/high blood pressure, cancer, diabetes, or who changed their diet because of high blood pressure did not yield a significant association (adjusted HR=0.83; 95% CI: 0.65–1.06; Table 2). None of the interaction terms were found to be statistically significant (*P* for interaction >0.05 for all). Further adjustment of our final model 3 for antioxidant micronutrients yielded a similar significant association.

## Discussion

In this prospective cohort study of nationally representative US adults, individuals with mushroom intake had lower risk of all-cause mortality than those without mushroom intake, independent of demographic, major lifestyle, and other dietary factors. Furthermore, we observed a dose-response relationship between greater mushroom intake and the risk of all-cause mortality. Replacing 1-serving of red or processed meat per day with 1-serving of mushrooms per day was associated with lower risk of all-cause mortality. We observed similar results to our final model 3 after further adjustment of antioxidant micronutrients.

These findings underscore the potentially important clinical and public health implications of mushroom consumption in the prevention of premature mortality. According to a previous study, consuming 1-serving of mushrooms per day instead of 1-serving of processed meats, poultry, and fish per day was associated with a lower risk of type 2 diabetes [25]. Consistent with a very recent systematic review and meta-analysis of observational studies, higher mushroom consumption was associated with a lower risk of total cancer, which could improve survivorship [47]. A study conducted by Zhang and colleagues showed that greater mushroom consumption was associated with a lower risk of dementia in elderly Japanese [20]. Furthermore, a study conducted in Singapore showed that mushroom intake was associated with lower odds of mild cognitive impairment [19]. However, several other epidemiological studies yield non-significant associations [21–25]. Furthermore, a previous systematic review and meta-analysis of fruit and vegetable intake and risk of mortality using 2 studies found no association between mushroom consumption and all-cause mortality [26]. The lack of observed significant associations between mushroom consumption and cause-specific mortality in our study could be due to the lack of statistical power caused by small sample sizes. Furthermore, we did not observe a significant association after excluding participants with major chronic conditions, which could due to the lack of sufficient power caused by the exclusions.

The potential biological mechanisms underlying the association between mushroom consumption and a lower risk of all-cause mortality may stem from their natural antioxidant properties due to specific mushroom components ergothioneine and glutathione. Oxidative stress occurs as a result of the imbalance between pro-and antioxidants defense systems, which has been associated with the etiology and pathogenesis of many chronic diseases that currently account for a vital portion of death [48, 49]. Because of the important role of oxidative stress in the development of many chronic diseases, antioxidants may play a significant role in the prevention of chronic diseases and the risk of premature death. Mushrooms are a potent source of powerful antioxidants and are therefore more likely to lower oxidative stress induced by reactive oxygen species [13]. The importance of mushrooms as a source of dietary ergothioneine stems from their unique role in ergothioneine biosynthesis and resulting high levels of this antioxidant Ergothioneine has been proposed as a “longevity vitamin” stemming from its many important functions (eg., antioxidant, cytoprotective, and anti-aging) in the human body [50]. Consistently, a recent unbiased plasma metabolomics study identified ergothioneine as the major metabolite associated with a health-conscious food pattern and reduced risk of cardiometabolic disease and mortality [51]. Another recent review study also proposed that ergothioneine could be used as a therapeutic to reduce the severity and mortality of coronavirus infectious disease 2019 (COVID-19) [52]. Despite the benefits of ergothioneine, its consumption remains relatively low in the US. It is estimated that the US has the lowest estimated average ergothioneine consumption (mg/day) compare to other industrialized countries such as Italy, Ireland, France, and Finland [53]. Mushrooms also contain other bioactive compounds including fiber-associated monosaccharides, chitin, and β-glucans [2]. Previous researchers demonstrated that glutathione levels are high in most mushroom species compared to any other vegetables or fruit [13].

Mushrooms are also low in energy, sodium, fats, and high in fiber, vitamins, and minerals (e.g., selenium and copper) and play an important role in a healthful diet [2–5]. An analysis of continuous NHANES data from 2001 to 2010 revealed that mushroom consumption is associated with better nutrient intake and diet quality as measured by the HEI-2005 in US adults [34]. Compared with individuals who were non- mushroom consumers, those who were consumers had higher intakes of protein, thiamin, selenium, copper, and folate, and lower intake of total and added sugars.

In the current *2015–2020 Dietary Guidelines*, mushrooms are categorized in “other vegetable”, which has a recommended intake of 4 cups per week [54]. Previous studies have suggested the establishment of a third (fungi) food kingdom or at least to raise awareness about the potential health benefits of mushroom consumption [1, 2]. However, efforts in this regard have been hampered by the relative lack of large-scale observational studies regarding the association between mushroom intake and major health outcomes.

The strengths of the current study include the large sample size of nationally representative US adults and the long duration of follow-up (up to 27 years). To the best of our knowledge, this is the first prospective cohort study to use NHANES data to examine the association between mushroom consumption and risk of all-cause and cause-specific mortality among US adults. Our results are robust to adjustment for a wide range of potential confounders, substitution effect of mushroom, and the propensity score analysis.

The study has some limitations that need to be addressed. First, mushroom consumption was assessed only at baseline using a single 24-h recall data. We did not have repeated measures data to examine the association of long-term pattern of mushroom consumption with risk of mortality. In addition, a single 24-h recall may not have adequately captured the within-person variation in mushroom intake. Such nondifferential measurement error may have underestimated the association between mushroom intake and risk of mortality. Second, because we used the USDA food codes for recipe foods to identify mushroom intake, the misclassification of exposure to mushrooms is likely inevitable. This includes misclassification of mushrooms as a vegetable and inaccurate assessment of mushroom content in the mixed foods. Third, information on the different types of mushrooms was not available, and, therefore, we may have missed the effect of particular mushrooms on mortality risk. Fourth, this was an observational study, and thus it is not possible to conclude that the inverse association between mushroom intake and mortality reflects cause and effect and should not be directly interpreted as evidence of causal relationships without considering other lines of evidence [55]. In addition, the NHANES III nutrients database does not contain information on ergothioneine and glutathione intake, therefore we could not include these variables in the present analysis. Lastly, even though we controlled for major potential confounders including, demographics, major lifestyle, and dietary risk factors in the models, residual confounding is possible in observation studies. Despite the aforementioned limitations, this study provides important information regarding the potential protective effects of mushrooms in lowering the risk of premature mortality among American adults.

## Conclusions

The current study showed a significant inverse association between mushroom consumption and risk of all-cause mortality. Our findings may provide evidence to support public health recommendations to increase awareness about the health-promoting effects of mushrooms. More prospective cohort studies are needed to further replicate our finding and clarify the potential role of mushroom intake in lowering the risk of mortality.

## Supplementary Information

**Additional file 1: Supplemental Table 1.** Foods with Mushrooms identified by USDA food code in dietary recall, NHANES III 1988–1994.

## Data Availability

The datasets used and/or analyzed during the current study are available at https://wwwn.cdc.gov/nchs/nhanes/nhanes3/DataFiles.aspx

## Abbreviations

NHANES: National Health and Nutrition Examination Survey
NCHS: National Center for Health Statistics
CDC: Centers for Disease Control and Prevention
SNDB: Survey Nutrient Database System
DDC: Dietary Data Collection
ICD-9: The Ninth Revision of the International Classification of Diseases
ICD-10: The Tenth Revision of the International Classification of Diseases
HRs: Hazard ratios
CIs: 95% confidence intervals
SE: Standard errors
US: United States
USDA: U.S. Department of agriculture
HEI-2000: Healthy Eating Index-2000
IFF: Individual Foods File
g: Grams
NDI: National Death Index
