# Supplementary material for: Association of mushroom consumption with all-cause and cause-specific mortality among American adults: prospective cohort study findings from NHANES III
Source: Nutr J. 2021 Apr 22;20:38. doi: 10.1186/s12937-021-00691-8 (PMC8061446; doi:10.1186/s12937-021-00691-8)
Supplement: Supplementary file 1 — Additional file 1: Supplemental Table 1. Foods with Mushrooms identified by USDA food code in dietary recall, NHANES III 1988–1994. [file 12937_2021_691_MOESM1_ESM.docx]

**Supplemental Table 1**. Foods with Mushrooms identified by USDA food code in dietary recall, NHANES III 1988-1994.

| **USDA food code** | **USDA food descriptions** |
| --- | --- |
| 7521901 | Mushrooms, cooked, NS as to form, fat not added in cooking |
| 3210513 | Egg omelet or scrambled egg, with onions, peppers, tomatoes, and mushrooms |
| 7521902 | Mushrooms, cooked, NS as to form, fat added in cooking |
| 2735041 | Tuna noodle casserole with vegetables and (mushroom) soup |
| 7560703 | Mushroom soup, canned, undiluted |
| 2725063 | Tuna noodle casserole with (mushroom) soup |
| 7220202 | Broccoli casserole (broccoli, rice, cheese, and mushroom sauce) |
| 7220124 | Broccoli, cooked, NS as to form, with mushroom sauce |
| 7560701 | Mushroom soup, cream of, prepared with milk |
| 7521900 | Mushrooms, cooked, NS as to form, NS as to fat added in cooking |
| 2751026 | Cheeseburger, 1/4 lb meat, with mushrooms in sauce, on bun |
| 2724225 | Chicken or turkey and noodles with (mushroom) soup (mixture) |
| 7560702 | Mushroom soup, cream of, prepared with water |
| 2714400 | Chicken or turkey with (mushroom) soup (mixture) |
| 2741420 | Beef with vegetables (excluding carrots, broccoli, and dark-green leafy (no potatoes)), (mushroom) soup (mixture) |
| 7541402 | Mushrooms, stuffed |
| 2731531 | Beef, rice, and vegetables (including carrots, broccoli, and/or dark-green leafy), (mushroom) soup (mixture) |
| 7560704 | Mushroom soup, with meat broth, prepared with water |
| 2734542 | Chicken or turkey, rice, and vegetables (excluding carrots, broccoli, and dark-green leafy), (mushroom) soup (mixture) |
| 2731532 | Beef, rice, and vegetables (excluding carrots, broccoli, and dark-green leafy), (mushroom) soup (mixture) |
| 2731162 | Beef, potatoes, and vegetables (excluding carrots, broccoli, and dark-green leafy), (mushroom) soup (mixture) |
| 2745051 | Tuna casserole with vegetables and (mushroom) soup, no noodles |
| 7534014 | Vegetable combination (green beans, broccoli, onions, mushrooms), cooked, fat not added in cooking |
| 5830405 | Spaghetti with meat and mushroom sauce (diet frozen meal) |
| 2725071 | Tuna and rice with (mushroom) soup (mixture) |
| 7560713 | Mushroom soup, made from dry mix |
| 2831602 | Beef and mushroom soup, canned, low sodium |
| 2731331 | Beef, noodles, and vegetables (including carrots, broccoli, and/or dark-green leafy), (mushroom) soup (mixture) |
| 2734348 | Chicken or turkey, noodles, and vegetables (excluding carrots, broccoli, and/or dark-green leafy), cream, white, or (mushroom) soup-based sauce (mixture) |
